# Infective endocarditis in Principal Hospital of Dakar: a retrospective study of 42 cases over 10 years

**DOI:** 10.11604/pamj.2017.26.40.10020

**Published:** 2017-01-30

**Authors:** Djibril Marie Ba, Mouhamed Cherif Mboup, Nafissatou Zeba, Khadidiatou Dia, Awa Ndaw Fall, Fatou Fall, Pape Diadie Fall, Sara Boury Gning

**Affiliations:** 1 Department of Cardiology, Principal Hospital of Dakar, Dakar, Senegal; 2 Department of Internal Medicine, Principal Hospital of Dakar, Dakar, Senegal

**Keywords:** Endocarditis, heart valves, fever, Dakar

## Abstract

Infective Endocarditis (IE) is an endocardial infection usually caused by bacteria that affects not only the native heart valves but also, with increasing frequency intravascular implanted devices and congenital heart diseases. Despite medical advances, IE remains a life-threatening disease with substantial morbidity and mortality. In Africa, its diagnosis and treatment are still a major challenge in clinical practice. The objective of this work was to study the epidemiological, clinical features, diagnostic techniques currently used in medical practice and the range of micro-organisms that are responsible. This was a retrospective study done at Principal Hospital of Dakar. We include all patients who were admitted with clinical manifestations of definite or possible IE according to the extended DUKE criteria between January 1^st^, 2005 and December 31^st^, 2014. We collected and analyzed epidemiological, clinical, paraclinical and outcomes data of 42 patients. Hospital prevalence of IE was 0.078% (42/53711). The mean age was 27.5+/- 18 years with a sex ratio (M/F) of 0.55. IE were more common in patients with damaged or abnormal heart valves (78.6%) and in thoses with underlying structural defects (14.3%). The most common presenting symptoms were fever (90%) and cardiac murmurs (81%). Extracardiac clinical manifestations were very rare. The usual laboratory parameters of inflammation were elevated in 90% of patients. Blood cultures were negative in 50% of cases and positive in 21.4%. The main organism found was Staphylococcus aureus. Echocardiography found vegetations in 95.2% of cases, chamber enlargement in 73.8% and mitral regurgiation in 83.3%. Broad-spectrum penicillins including ampicillin and gentamycine were used for all patients. Major complications were heart failure (47.6%). Strokes and cerebral abcess (23.8%) and Vascular emboli 14.3%. Hospital mortality was 31%. IE remains a life-threatening disease with hight mortality despites improved techniques of diagnosis and modern antibiotics.

## Introduction

Infectious endocarditis (IE) denotes infection of the endocardial surface of the heart and implies the physical presence of microorganisms in the lesion. Although the heart valves are most commonly affected, the disease may also occur within septal defects or on the mural endocardium but also on intavasculary implanted foreign materials such as valvular prostheses or pacemaker electrodes. Despite medical advances, IE remains a life-threatening disease with substantial morbidity and mortality. Its diagnosis and treatement are still a major challenge in clinical practice. In developped countries, Chronic rheumatic valvular disease has been supplanted by mitral valve prolapse with mitral regurgitation and degenerative aortic valve disease as the leading cardiac conditions predisposing to bacterial endocarditis in adults [1]. Nosocomial endocarditis associated with therapeutic interventions such as implanted devices, dialysis shunts is increasing in frequency. Many studies found that Staphylococcus aureus was the most common pathogen throughout much of the world [2]. In Sub-Saharan Africa, IE is recongized as a disease associated with rheumatic heart disease. Although many factors have altered the epidemiology of IE while maintaining its incidence: an aging population with degenerative valvular disease, increasing number of valve replacements and medical intervention [3]. The objective of this work was to study the epidemiological, clinical features, diagnostic techniques currently used in medical practice and the range of micro-organisms that are responsible.

## Methods

This was a retrospective study conducted at the cardiology, paedriatrics, Internal medicine, Infectious diseases and Intensive Care Unit (ICU) Departments of Principal Hospital of Dakar over a period of 10 years (January 1^st^2005 to December 31^st^ 2014). We include all patients who were admitted with manifestations of definite or possible IE according to the extended DUKE criteria [4]. We collected and analyzed epidemiological, clinical, paraclinical and outcomes data of patients. We studied data on age, gender, past history including history of medical interventions, predisposing heart disease and underlying disease. We sought the presence of fever, cardiac murmur and extracardiac symptoms of EI such vascular and immunological phenomena (Osler's nodes, Janeway lesions, splinter hemorrhages etc.). All patients had a complete physical examination and a laboratory assessment. Blood cultures were done for most of patients and the other tests included urine examination, renal function and the usual laboratory parameters of inflammation such as C-Reactive Proteine, Erythrocyte sedimentation rate and fibrinogen. On the ECG we looked for chamber enlargement, rhythm and conduction abnormalities. Doppler echocardiography were performed for all patients for the demonstration of endocardial involvement and the assessment of left and right ventricular function and tissue destruction. Current aspects of antibiotics therapy were evaluated as well as evolution during hospitalization. The studied parameters were entered into an electronic questionnaire using Epi Iinfo version 6.0 of the World Health Organization. Data analysis was performed using SPSS (Statistical Package for Social Sciences). Quantitative data were expressed as mean± standard deviation. Qualitative data were expressed as percentage.

## Results

We included 42 patients. Hospital prevalence of IE was 0.078% (42/53711). 27 patients (64.29%) were females and 15 patients (35.71%) were males giving a sex ratio (M/F) of 0.55. The mean age of patients was 27.5+/- 8 years with a range of 8 months and 72 years. Most of patients (54.76%) was under 25 years. Most cases were encountered in the department of cardiology (54.8%), paedriatrics (26.2%) and ICU (11.9%). The Diagnosis latency ie, the interval from the onset of symptoms to the definite diagnosis of IE was 29.97 days. Predisposing conditions to IE in our patients (Table 1) were dominated by rheumatic valvular disease (64.3%). Six patients had endocarditis previously, 4 patients had a valvular prosthesis (9.5%) and 6 patients had congenital heart defects (14.3%). Predominant causes of bacteremia that were found in 27 patients were: dental caries (26.2%), urosepsis (26.2%), skin infections (14.2%) and Ear Nose Throat (ENT) infections (9.5%). For other patients no cause was found.

**Table 1:** Predisposing conditions to Infective endocarditis

Predisposing conditions	number	Percentage
Rheumatic heart valves	27	64.3%
**Congenital heart defects**		
Tetralogy of Fallot	1	2.4%
ventricular septal defect	3	7.1%
Atrio-ventricular septal defect	1	2.4%
Patent ductus arteriosus	1	2.4%
Degenerative valve disease	2	4.8%
**Valvular prosthesis**		
mitral	3	7.1%
aortic	1	2.4%
Other (diabete, hodgkin, Basedow, HIV)	4	9.5%

The most common presenting symptoms were fever (90%), cardiac murmurs (81%) and dyspnea (42.9%). The fever was above 38°5C in 86.8% of cases with high remitting pyrexia often associated with chills and night sweats in 6 patients. Weight loss was found in 22 patients (52.4%). Extracardiac clinical manifestations were dominated by vomiting (26.2%), abdominal pains (14.28%) and seizures (7.14%). Clubbing was found in 2 patients and 1 patient had splenomegaly. Mild to moderate anemia was commonly present (82.1%) with a hypochromic, microcytic picture. Neutrophil Leukocytosis was common (90%) and the Erythrocyte sedimentation rate (ESR) and C-reactive proteine (CRP) were elevated in 90% of patients. Renal function test results showed an elevated creatinine level in 17.1% of patients, proteinuria in 12% and microscopic hematuria in 1 patient. Blood cultures were negative in 50% of cases and positive in 21.4% of cases. The main organism found were Staphylococcus aureus (55.5%), Enterococcus faecalis (22.2%), Klebsiella pneumoniae (11.1%) and Escherichia coli (11.1%) Table 2. Echocardiography found vegetations in 95.2% of cases, Chamber enlargement in 73.8% and mitral regurgiation in 83.3%.

**Table 2:** Positive blood cultures

Micro-Organisms	Number	Percentage
Staphylococcus aureus	5	55.5%
Enterococcus faecalis	2	22.2%
Klebsiella pneumoniae	1	11.11%
Escherichia coli	1	11.11%

The mitral and aortic valves, in respectively 23 patients (57.5%) and 8 patients (20%), were the most frequent affected sites; there was 1 case (2.5%) of tricuspid valve endocarditis, and 1 patient (2.5%) had two valves affected; there was 3 cases (7.5%) of septal defects endocarditis and 2 cases(5%) were prosthetic valve infections. There was 4 cases of rupture of chordae. Concerning treatment, antibiotics, administred parenterally in sufficient doses, were used in 41 patients (97.6%). The average duration of antimicrobial treatment was 17.48 days. Penicillins (68.2%), cephalosporins (68.2%), aminoglycosides (87.8%) and glycopeptides were the most used. No surgical treatment was performed.

The evolution during hospitalization after a mean hospital stay of 21.45 days was favorable in 28 patients (66.6%). Complications were dominated by Congestive heart failure (CHF) (47.6%), neurological complications (31%) and major systemic emboli in 14.3% of cases. The complications are summarized in Table 3. Thirteen (13) deaths (31%) were recorded, 8 of which were females (61.5%) and 5 were males(38.4%).

**Table 3:** Complications

Complications	Number	Percentage
heart failure	20	47.6%
**Neurologic**		
Stroke	6	14,3%
Cerebral abscess	4	9.5%
Meningitidis	1	2.4%
Cerebral hemmorrhagies	2	4.8%
Acute renal failure	2	4.8%
**Embolic events**		
Splenic infarction	3	7.1%
Renal infarction	1	2.4%
Femoral artery emboli	2	4.8%
Septic arthritis	1	2.4%

In patients with negative blood cultures, mortality was 69.2% against 30.8% in patients with positive blood cultures. Mortality according causative organisms was 50% for Staphylococcus aureus, 25% for Enterococcus faecalis and Klebsiella pneumoniae respectively. Ten (10) deaths were due to CHF, 2 deaths to neurological complications (meningitidis and cerebral abscess) and 1 death was due to anaphylactic reaction following blood transfusion.

## Discussion

In our study the prevalence of IE was 0.078%. In Africa, different series show a higher prevalence around 1.6% [5, 6]. This difference is difficult to explain because criteria for diagnosis and methods of reporting vary with different series. Also, in our study all cases were definites IE, while in other series, only a small proportion of clinically diagnosed cases are categorized as definite. HASE [7] in Japan found a prevalence of 0.19% over 12 years-study. In France its incidence (0.0032%) has not changed appreciably over the past years according to REVEST's study [8]. These low incidences are probably due to a decline in the incidence of rheumatic heart disease and antimicrobial prophylaxis before selected dental and invasive procedures in most developped countries. However, the use of antibiotics for IE prophylaxis in procedures that can cause bacteremia is controversial. The mean age of our patients was 27 years. Our study confirms young age of patients with IE as has been emphasized in previous African works. IKAMA [9] and NEBIE [5] found, respectively, a mean age of 30.6 years and 32.6 years. In European an American series, patients with IE are typically older with a mean age around of 60 years [10]. A number of factors may relate to this difference in age distribution. First, there has been a change in the nature of the underlying heart disease owing to a decline in the incidence of rheumatic heart disease. Second, the age of the population has been steadily increasing, and people with rheumatic or congenital heart disease are surviving longer. In addition, such patients increasingly undergo prosthetic valve surgery, an important risk factor for IE [11–13]. Our study confirms female predominance as has been emphasized in previous african works [9, 14]. This female predominance relate to the higher incidence of rheumatic disease in women as noted in African literature [15–17].

In Europe and America men are more commonly affected than women with a sex ratio up to 2. This is close to what is found in some africans country like Tunisia [18]. Predisposing conditions to IE in our patients were dominated by rheumatic valvular disease (64.3%). Indeed, most of Africans series on the occurence of IE, have rheumatic valve as a dominant underlying disease [14, 19]. In high income countries, currently, patients with degenerative valve disease, congenital heart disease, implantable devices and users of illicit intravenous drugs rather than those with rheumatic heart disease, account for the majority of cases of IE [10, 20, 21]. Predominant causes of bacteremia that were found in our patients were: dental caries (40.7%), urosepsis (40.7%). Dental carries as a cause of bacteremia is more common in african series than urosepsis [9, 19]. In general, risk of IE is considered to be highest for oral or dental procedures in which the oral mucosa is penetrated and in which gingival or mucosal bleeding is likely to occur. The risk of bacteremia is substantially lower for invasive genitourinary procedures. Clinically, IE in Africans does not appear much different from that of high income countries. This has been emphasized by several authors [8, 14, 22, 23]. The picture of IE is dominated by fever and cardiac murmurs, found in our study, respectively in 90% and 81% of cases. Blood cultures remain the definitive procedure for diagnosing IE. Blood cultures were negatives in 50% of cases in our study. Similar findings were noted by LAKHDHAR and YAMEOGO [19, 24] versus 5% found by HASE in Japan [7] and 9% by HOEN in France [25]. These high rates of negative blood cultures in african series may relate to prior antibiotic therapy and also by the method employed.

IE is usually caused by Stapylococcus aureus. This has been emphasized by several authors both in Africa and Europa [19, 25, 26]. In our study, Staphylococcus aureus was the main pathogen and was responsible of 50% of death. This result confirms data from the literature. The utility of other blood laboratory tests in the diagnosis of IE is limited. Hematologic parameters are often abnormal in IE, but none is diagnostic. Anemia is nearly always present and usual parameters of inflammation elevated.

Echocardiography has become paramount in the process of evaluating IE. It is crucial in detecting vegetations, echogenic distinct masses from the adjacent valve with independent motion from the valve itself. In our study, vegetations were found in 95.2% of cases. This percentage is close to what is found in Africa, Japan and France [7, 8, 19]. As in many african studies [17, 19], mitral valves (57.5%) were the most frequently affected sites weither it is the aortic valves in occidental series [8].

Following the establishment of a diagnosis using clinical, microbiological, and echocardiographic methods, antibiotics should be administered in a dosage designed to give sustained bactericidal serum concentrations throughout much or the entire dosing interval. A prolonged course of therapy is necessary to eradicate microorganisms growing in valvular vegetations [27]. In our study, almost all patients was under antibiotics. Penicillins and aminoglycosides were the most used. Duration of treatment was shorter than indicated. The reasons for this were due to many factors including lack of avalaibility and inability of some patients to meet the high cost of certain antibiotics. No surgical treatment was performed in our study as in many other african series while it has become an important adjunct to medical therapy in the management of IE and is now used in at least 25% of the cases in occident [8].

Favorable outcomes, as seen in our work and as noted in literature, seems higher in patients with positive blood cultures than those with negative ones. As usual in african series [14, 19, 28], complications were dominated by cardiac failure (47.6%) and was responsible of 76.9% of death. Neurologic manifestations occur in 20% to 40% of the cases and may dominate the clinical picture, especially in staphylococcal IE. Stroke is the most common neurologic complication of IE, occurring in 9.6% of patients [29, 30]. In our work stroke was present in 14.3% of cases. The development of clinical neurologic deterioration during IE is associated with a two- to fourfold increase in mortality. Less common noted in literature and found in our study were 4 cases of cerbral abscess (9.5%) and 1 case of meningitidis [31, 32]. Mortality remains high in Africa around 31% [23, 24].

## Conclusion

Because of the variability in clinical presentation and the heterogeneity in both microbiologic etiology and the patient population, IE remains a challenging disease for clinicians. In Africa, where most health facilities are understaffed, the morbidity and mortality of IE remains high due to many reasons including inability to meet high cost of antibiotics and cardiac surgery which are rarely available. In addition, the costly treatment presents a huge financial burden on both the patients and the health system. Therefore, antimicrobial prophylaxis before selected dental and invasive surgical and diagnostic procedures should become standard and routine.

### What is known about this topic

- In Sub-Saharan Africa, IE is recongized as a disease associated with rheumatic heart disease and Staphylococcus aureus is the main pathogen;
- Patients affected are young with a female predominance;
- complications are dominated by congestive heart failure and mortality remains very high.

### What this study adds

- The Diagnosis latency IE, the interval from the onset of symptoms to the definite diagnosis of IE remains long (29.97 days);
- Favorable outcomes, seems higher in patients with positive blood cultures than those with negative ones which represent about 50% of cases;
- Despite medical advances, No surgical treatment was performed in our study while it has become an important adjunct to medical therapy in the management of IE.
